# Advances in Atomic Layer Deposition (ALD) Nanolaminate Synthesis of Thermoelectric Films in Porous Templates for Improved Seebeck Coefficient

**DOI:** 10.3390/ma13061283

**Published:** 2020-03-12

**Authors:** Xin Chen, Helmut Baumgart

**Affiliations:** 1 Department of Electrical and Computer Engineering, Old Dominion University, Norfolk, VA 23529, USA; xchen011@odu.edu; 2 Applied Research Center at Thomas Jefferson National Accelerator Laboratories, 12050 Jefferson Avenue, Suite 721, Newport News, VA 23606, USA

**Keywords:** thermoelectric, lead chalcogenide, PbTe, PbSe, nanolaminates, Seebeck coefficient, atomic layer deposition

## Abstract

Thermoelectrics is a green renewable energy technology which can significantly contribute to power generation due to its potential in generating electricity out of waste heat. The main challenge for the development of thermoelectrics is its low conversion efficiency. One key strategy to improve conversion efficiency is reducing the thermal conductivity of thermoelectric materials. In this paper, the state-of-the-art progresses made in improving thermoelectric materials are reviewed and discussed, focusing on phononic engineering via applying porous templates and ALD deposited nanolaminates structure. The effect of nanolaminates structure and porous templates on Seebeck coefficient, electrical conductivity and thermal conductivity, and hence in figure of merit zT of different types of materials system, including PnCs, lead chalcogenide-based nanostructured films on planar and porous templates, ZnO-based superlattice, and hybrid organic-inorganic superlattices, will be reviewed and discussed.

## 1. Introduction

### 1.1. Introduction to Thermoelectrics

**Figure 1:**
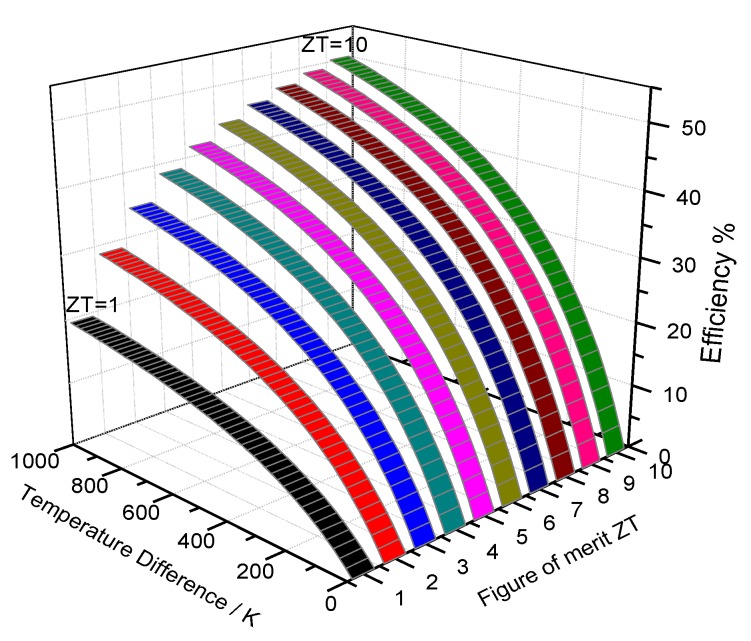
Simulation results of the dependence of efficiency on figure of merit ZT of thermoelectric (TE) device and temperature difference [1].

Thermoelectric (TE) materials have attracted much attention for their potential in the conversion of waste heat to electricity, and consequently could be used for heating and cooling systems. As a green renewable energy technology, thermoelectrics has been applied in TE power generators to use electricity generated from waste heat as a power source for automobiles or even spacecraft and satellites, and in thermoelectric coolers, taking advantage of its small size, flexible shape, portability and long lifetime. The challenge for the current state-of-the-art TE devices is the low conversion efficiency, which is true for all green renewable techniques. The efficiency of a thermoelectric device is expressed as:

(1) $$\eta =\frac{T_{H}-T_{C}}{T_{H}}\left[\frac{{\left(1+ZT\right)}^{1/2}-1}{{\left(1+ZT\right)}^{1/2}+\left(T_{C}/T_{H}\right)}\right]$$

where$T_{C}$ and $T_{H}$ indicate the temperature of the cold and hot side. The conversion efficiency η is determined by the dimensionless thermoelectric figure of merit ZT,

(2) $$Z\bar{T}=\frac{{\left(S_{p}-S_{n}\right)}^{2}\bar{T}}{{\left[{\left(\rho_{n}\kappa_{n}\right)}^{1/2}+{\left(\rho_{p}\kappa_{p}\right)}^{1/2}\right]}^{2}}$$

where S, ρ, κ are Seebeck coefficient, electrical resistivity and thermal conductivity, respectively. Subscript n and p denote properties for n-type and p-type of thermoelectric materials in TE devices. $\bar{T}$ is the average temperature between the hot and code surfaces. Figure 1 shows the simulation result for the dependence of the conversion efficiency η on ZT of TE devices and temperature difference. The higher the figure of merit ZT, the higher the conversion efficiency η would be. For current state-of-the-art TE devices, the figure of merit ZT value is usually around one, which translates into a conversion efficiency that is usually no higher than 10%. For large-scale practical applications, it is critical to synthesize better TE materials with a higher figure of merit zT, in order to promote attractive conversion efficiencies for TE devices. 

zT of thermoelectric materials is expressed as:

zT = S^2^σT/ (κ_e_+κ_L_) (3)

where S is the Seebeck coefficient, σ is the electrical conductivity, T is the temperature in Kelvin, $\kappa_{e}$ is the thermal conductivity due to electrons, and $\kappa_{L}$ is lattice thermal conductivity due to phonons [2]. To increase the zT value of TE materials, it is not sufficient to simply increase the Seebeck coefficient (S) and the electrical conductivity (σ), and to decrease the thermal conductivity ($\kappa_{e}$ and $\kappa_{L}$), since these parameters are correlated to each other in a complex way. Bulk thermoelectric materials are interrelated to such a degree as to make independent control of these variables to increase zT almost impossible, because an increase in S results in a decrease in σ resulting in practically zero gain. The Seebeck coefficient can be expressed by the Mott relation:

(4) $$S=\frac{8\pi^{2}k_{B}^{2}}{3eh^{2}}m^{*}T{\left(\frac{\pi }{3n}\right)}^{3}$$

where $k_{B}$ is the Boltzmann constant, $m^{*}$ is the effective mass, h is the Planck constant, and n is the charge carrier density. The Seebeck coefficient is inversely proportional to the density of charge carriers. An increase of the Seebeck coefficient S leads to a reduction of electrical conductivity σ and carrier density, while enhancement of electrical conductivity σ results in an increase of thermal conductivity $\kappa_{e}$ according to the Wiedemann–Franz law in Equation (5) [3]. High Seebeck coefficients are usually found in semiconductor or insulator materials with lower carrier density. High electrical conductivity is typically observed in metals, and consequently the optimum power factor (S^2^σ) can be found in highly doped semiconductors with a carrier concentration of 10^−19^ ~ 10^−21^ cm^3^ [3]. Since κ is inversely proportional to zT, low thermal conductivity is the other essential requirement for high zT TE materials. The thermal conductivity of TE materials consists of two components: the electronic thermal conductivity $\kappa_{e}$, which results from heat carrying charge carriers (electrons or holes), and the lattice thermal conductivity $\kappa_{L}$, which results from lattice vibrations through crystal lattices. The Wiedemann–Franz law provides the relationship between electrical conductivity and electronic thermal conductivity $\kappa_{e}$, which is described by Equation (5) (equivalent to Equation (7)):

(5) $$\kappa_{e}=L_{o}\sigma T$$

(6) $$L_{o}={\left(\frac{k_{B}}{e}\right)}^{2}\left(\frac{\pi^{2}}{3}\right)$$

(7) $$\kappa_{e}=ne\mu {\left(\frac{k_{B}}{e}\right)}^{2}\left(\frac{\pi^{2}}{3}\right)T$$

where $L_{o}$ described by equation 6, is the Lorentz number with a value of 2.44 × 10^−8^ W-Ω/K^2^. Here $k_{B}$ is the Boltzmann constant, n is the carrier density, μ is the carrier mobility, and σ is the electrical conductivity. The Wiedemann–Franz law provides the ratio of the electrical component of thermal conductivity and the electrical conductivity in relation to the product of Lorenz number ($L_{o}$) and temperature (T) [4]. This law is applicable at high temperatures and at low temperatures, whereas it fails at intermediate temperatures. In metals and highly doped semiconductors, this ratio is almost a constant at a given temperature. However, in semiconductors, this ratio is much greater, which makes it more suitable for thermoelectric applications. The electronic thermal conductivity $\kappa_{e}$ is proportional to the electrical conductivity. However, it may not be feasible to achieve high zT by reducing the electronic thermal conductivity, because the reduction in electronic thermal conductivity $\kappa_{e}$ will directly affect the electrical conductivity σ and will ultimately decrease the power factor ($S^{2}\sigma$). The lattice thermal conductivity $\kappa_{L}$ describes the thermal conductivity due to heat transfer by way of elastic vibrations of lattices, which is defined by:

(8) $$\kappa_{L}=(1/3)C_{\nu }\nu_{s}\lambda_{ph}$$

where $C_{\nu }$ is heat capacity, $\nu_{s}$ is sound velocity, and $\lambda_{ph}$ is the mean free path of phonons. The lattice thermal conductivity $\kappa_{L}$ is the only parameter that is independent of the Seebeck coefficient S, the electrical conductivity σ or the electronic thermal conductivity $\kappa_{e}$. Therefore, reduction in lattice thermal conductivity is a feasible and effective approach to enhance zT.

### 1.2. Approaches for Thermoelectrics Improvement

**Figure 2:**
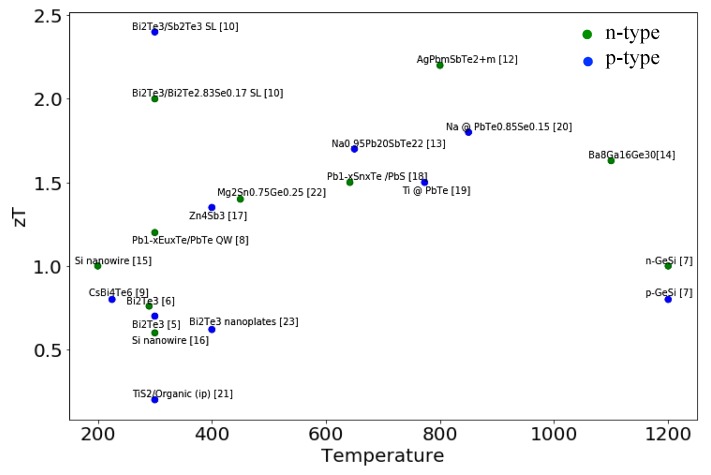
Major milestones achieved for zT as a function of temperature. p-type Bi_2_Te_3_ [5], n-type Bi_2_Te_3_ [6], GeSi [7], Pb_1-x_Eu_x_Te/PbTe quantum well [8], CsBi_4_Te_6_ [9], p-type Bi_2_Te_3_/Sb_2_Te_3_ SL [10], n-type Bi_2_Te_3_/Bi_2_Te_2.83_Se_0.17_ SL [10], n-type (Bi,Sb)_2_(Se,Te)_3_ Quantum Dot SL [11], AgPb_m_SbTe_2+m_ (m = 10,18) [12], Na_0.95_Pb_20_SbTe_22_ [13], Ba_8_Ga_16_Ge_30_ [14], Si nanowire [15,16], Zn_4_Sb_3_ [17], Pb_1-x_Sn_x_Te /PbS (x = 0.08) [18], Ti doped PbTe [19], Na doped PbTe_0.85_Se_0.15_ [20], TiS_2_/Organic (ip) [21], Mg_2_Sn_0.75_Ge_0.25_ [22], Bi_2_Te_3_ nanoplates [23].

For the development of thermoelectrics, bismuth telluride (Bi_2_Te_3_), lead chalcogenides like lead telluride (PbTe) and silicon germanium (SiGe) bulk materials are the first generation TE materials, mainly working in an optimal way at room temperature, intermediate temperature range and high temperature [24], respectively. Figure 2 shows the major milestones achieved for zT as a function of temperature. Great progress has been made in recent years. Most of the R&D efforts have achieved a zT value of 1 during the last two decades. The highest zT reported in Bi_2_Te_3_/Sb_2_Te superlattices, is 2.4 [10]. The periodicity of the superlattice was controlled and optimized to modulate the transport of electrons and phonons in Bi_2_Te_3_/Sb_2_Te superlattices. Despite such a singular high achievement, progress in TE materials has still been limited to practical applications. To enhance the TE conversion efficiency, researchers are focused on two main directions: increasing the power factor ($S^{2}\sigma$), and decreasing the thermal conductivity κ.

The phonon glass electron crystal (PGEC) is one of the most popular approaches applied for the enhancement of zT. PGEC stands for an ideal hypothetical material that possesses the low thermal conductivity of glass and high electrical conductivity of crystals, which was first proposed by G. A. Slack et al [25]. This provides a strategy for zT enhancement by optimizing the thermal and electrical conductivity of TE materials. Generally, the electrical conductivity of semiconductors can be improved by highly doping the materials to either n-type or p-type. The enhanced charge carrier density results in an improvement in electrical conductivity. Regarding thermal conductivity, thermal transport in crystalline dielectric solids or amorphous glasses is accomplished by lattice vibrations and interactions of phonons. The phonons are the quantum description of a collective vibration of a lattice consisting of atoms or molecules. The lifetime of free electrons is zero in most non-metallic materials, indicating that electrons have no contribution to heat transport. Therefore, the electronic thermal conductivity was not taken into consideration in this case. The wavelength of phonons in crystalline solids is distributed over a broad range. First, principle calculations reveal that the long-wavelength phonons contribute 95% to the total thermal conductivity in bulk materials. The existence of defects, voids and interface boundaries in the crystals is acting as scattering centers to block heat transfer through phonons, which leads to a reduction of thermal conductivity. Therefore, the PGEC concept suggests that an ideal TE material can be realized in a cage-like “open structured” compound as a host crystal, where heavy mass atoms are trapped inside, acting as scattering centers of phonons, to reduce thermal conductivity. These PGEC materials were observed experimentally in skutterudites and intermetallic clathrates. 

A skutterudite is a cubic crystal structure with Im$\bar{3}$ space group. This structure was typically in the family of (Co, Ni, Fe, Ir, Rh)(P, Sb, As)_3_, containing voids with large unit cells [26]. It has been reported that the thermal conductivity of skutterudite structures can be altered by inserting rare earth elements into the host structure. The infiltrated heavy rare earth atoms act as scattering sites generating a large scale of local anharmonic lattice vibrations. As a result, the thermal conductivity decreases because the lattice vibration propagation is disturbed, while little effect on the electrical conductivity has been seen. The phonon propagation can be suppressed up to 58% resulting from the lattice phonon scattering, caused by the large mass of inserted rare earth atoms [27]. Therefore, skutterudites with infiltrated filled voids have been reported as excellent thermoelectrical materials [28,29].

Intermetallic clathrates are another candidate for the PGEC structure, which consist of a nano-caged structure working in the same way as skutterudites. Decreasing thermal conductivity was also realized by inserting guest molecules into the host cage of intermetallic clathrates. The off-center oscillations of guest atoms inside the voids, which is also called “rattling” or “rattler”, suppress the heat flow, and thereby reduce the thermal conductivity of the materials to values as low as ~2 W/Km [30,31]. In contrast, the electrons can move freely and undisturbed along the crystalline structure of the host materials. The intermetallic clathrate is typically formed by Zintl compounds with cage frameworks of Si, Ge, and Sn, and buried cationic guests [32]. The non-stoichiometric clathrate generates charge carriers, and thereby behaviors like semiconductors. The carrier density can be modulated in a range of 10^−19^ to 10^−20^ per cm^3^, resulting in a decent electrical conductivity and simultaneously a high Seebeck coefficient [33,34]. 

Reduction of thermal conductivity is a key strategy for the enhancement of zT. The lattice thermal conductivity of each material is limited by the mean free path of the phonons, resulting from atomic substitution and nanostructures in alloys [35,36]. It is reported that every material has its well-defined minimum lattice thermal conductivity. The lowest lattice thermal conductivity can be reached when the characteristic length scale corresponds to the mean free path of phonons and is equal to or approaching their wavelength [35]. Slack et al. has pointed out that the lowest thermal conductivity of heavy element semiconductors will be in the range of 0.1 ~ 0.5 W/mK at room temperature, and PbTe can exhibit a thermal conductivity as low as 0.25 W/mK, for instance [35]. 

There are various ways to expand the limit of thermal conductivity. By introducing nanoparticles, nano-sized polycrystalline, and numerous interfaces into bulk TE materials, such nanostructures covering a range of length scales from 5 nm ~ 10 μm can effectively scatter the phonons in a broad range of mean free paths, and thereby reduce the lattice thermal conductivity breaking the alloy limit. For instance, it is reported that compacted 30 ~ 50 nm-thick Bi_0.5_Sb_1.5_Te_3_ nano-platelets fabricated by hydrothermal method exhibit a low thermal conductivity of 0.37 W/mK at 295 K, which is much lower than the thermal conductivity of the parent bulk compounds (1.5~2.4 W/mK), and yields a zT value of 0.93 at 295 K [37]. Three years later, a joint research group from South Korea and the US reported on the same alloy of Bi_0.5_Sb_1.5_Te_3_, which was fabricated by using melted telluride to fuse bismuth, antimony, telluride granules, exhibiting a high zT of 1.86 at 320 K [38]. The granule size varied from 10 nm to 20 nm. The dense dislocation arrays generated at grain boundaries in the Bi_0.5_Sb_1.5_Te_3_ alloy played a significant role in scattering the phonons with mid-wavelength, resulting in a lower thermal conductivity of 0.33 W/mK at 320 K. 

**Figure 3:**
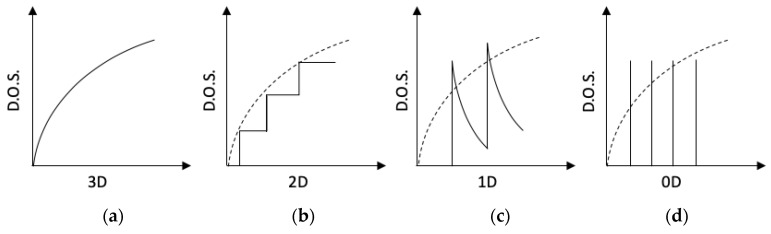
Electronic density of states as a function of energy for (**a**) 3-D bulk semiconductors, (**b**) 2-D quantum well or superlattices structures, (**c**) 1-D nanowire or nano-tube structures, and (**d**) 0-D quantum dots structures.

Nanostructuring is another proven approach to beat the alloy limit. In principle, all low dimensional nano-structures, including two-dimensional quantum wells or superlattices, one-dimensional nano-wires or nano-tubes, and zero-dimensional quantum dots, confine the motion of electrons in one or more dimensions, which decouples the dependence between the Seebeck coefficient *S*, the electrical conductivity σ and thermal conductivity κ, and make it possible to enhance the power factor product of ${\;S}^{2}\sigma$ and reduce κ somewhat independently [39]. This is attributed to the difference in the density of states of low dimensional materials and bulk materials. Figure 3 shows the density of states of semiconductors as a function of energy for 3-D material and low dimensional materials. As the dimensionality decreased from 3-D to 0-D, the dependence of the density of states on the energy changed from continuous in 3-D structures to step-like in 2-D structures and pulse-like in 1-D structures, and to discrete in 0-D structures. The electron confinement effect in low dimensional structures increased the density of states near the Fermi level, and thereby enhanced the electrical conductivity, since it is directly proportional to the density of states [40], under the assumption of energy independent electron scattering. Numerous novel innovative research approaches focused on low dimensional nanostructures [41], including quantum wells [10], quantum dots [42], and superlattices [11], in order to improve the figure of merit zT. P-type Bi_2_Te_3_/Sb_2_Te_3_ superlattices were found to exhibit the highest reported zT value of 2.4 at room temperature, with the lowest thermal conductivity of 0.22 W/mK, by adjusting the period of the superlattice to around 6 nm to control the transport of phonons and electrons in the superlattice [10]. Caylor and co-workers reported enhanced zT = 1.75 at 425 K, using PbTe/PbTe_0.75_Se_0.25_ quantum well structure with Se doping at 25% [43]. For 1-D nanowire structures so far, Bi based nanowires have been extensively investigated due to their properties at the transition between semi metal to semiconductor resulting from the size effect [39]. These nanowires are often synthesized using porous anodic aluminum oxide (AAO) or quartz (SiO_2_) templates. Bi nanowires with diameters ranging from 9~15 nm have been reported, with a factor of two more times enhancement in thermoelectric power factor S^2^σ, in the temperature range of 100~300K. An enormous zT enhancement was observed at a low temperature (~150K), which benefited from the reduction of the thermal conductivity. Herman and co-workers proposed a lead chalcogenide-based quantum dot superlattice system, PbTe/PbSeTe, embedding PbSe quantum dots in the PbTe matrix, which achieved a zT as high as 1.6 at 300K, with low thermal conductivity of 0.33 W/mK [11]. In addition, other quantum dot systems, such as AgPb_m_SbTe_20-m_ also known under the acronym LAST-18 [12], NaPb_m_SeTe_20-m_ known under the acronym SALT-20 [13], and (Pb_0.95_Sn_0.05_Te)_0.92_(PbS)_0.08_ [18], were reported to achieve zT values as high as 1.7 (at 700 K), 1.6 (at 675 K), and 1.5 at 650 K, respectively.

**Figure 4:**
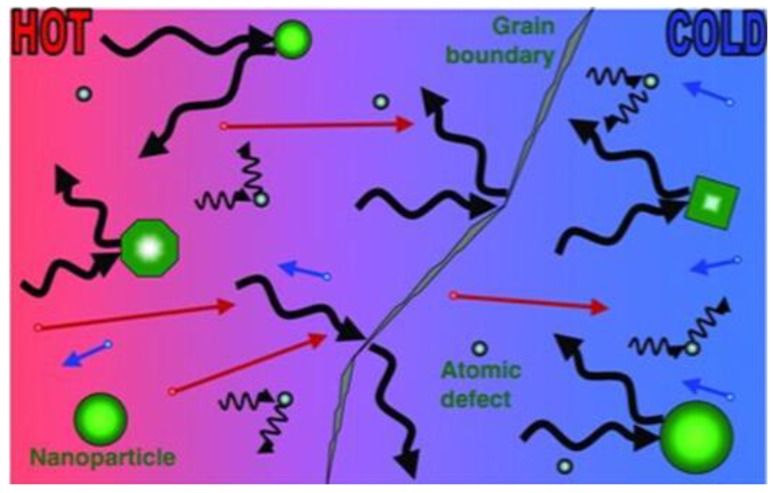
Schematic of phonon scattering mechanisms and electron transport within thermoelectric phonon glass electron crystal (PGEC) model material [44].

The reason why low dimensional nanostructures help to improve zT is attributed to the fact that the quantum confinement effect increases the density of states near the Fermi level, and consequently increases the thermal power factor ($S^{2}\sigma$). Additionally, the quantum confinement effect contributes to decouple the interdependence relationship between electrical and thermal properties, resulting in increasing electrical conductivity while reducing thermal conductivity quasi-independently. On the other hand, numerous interfaces in the lower dimensional structures result in enhanced phonon scattering, and consequently in a reduction of thermal conductivity and improved zT values. The latter is the dominant cause of zT enhancement [39]. Figure 4 displays the scattering mechanism of phonons and electron transport within TE materials. Since the mean free path of electrons is typically orders of magnitude smaller than that of phonons, the phonons could be scattered more efficiently, thereby blocking phonon propagation, while electron transport is not hindered. Meanwhile, phonons with a short wavelength can easily be scattered by atomic defects, while phonons with mid or long wavelength could be scattered by nanoparticles and boundary interfaces. By wisely selecting the dimension of nano-structuring, the phonon scattering would be enhanced efficiently, while the transport of electrons would not suffer. 

In this short review, we shall review the progress made in improving thermoelectric properties by implementing phononic engineering concepts, via applying porous templates and ALD deposited nanolaminate structures. This paper discusses the effect of ALD nanolaminate structures in conjunction with porous templates on Seebeck coefficient, electrical conductivity and thermal conductivity, and hence on the figure of merit, zT of PnCs, lead chalcogenide-based nanostructured films on planar and porous templates, ZnO-based superlattice, and hybrid organic-inorganic superlattices are discussed. Finally, the paper concludes with considerations on possible future work in this novel field. 

## 2. Atomic Layer Deposition Technique

For the deposition of nanostructured thin films, there are various techniques available, such as pulsed laser deposition (PLD) [45], metal-organic chemical vapor deposition (MO-CVD) [46], magnetron sputtering [47], molecular beam epitaxy (MBE) [48], electrochemical atomic layer deposition (E-ALD) [49] and atomic layer deposition (ALD) [50,51]. MBE is an ultra-high vacuum evaporation process at relative low temperature, which is advantageous for very-large-scale integration (VLSI) and is capable of generating complicated doped materials configurations with high purity. However, MBE suffers the drawback of high cost and very low throughput, which is incompatible with high volume production. E-ALD, which is also referred to as electrochemical atomic layer epitaxy (ECALE) or underpotential deposition (UPD), is an atomic layer deposition method in an electrochemical environment, in which the atomic monolayers are formed by repeatedly exposing substrates to proper chemical solutions with proper potentials. E-ALD has a number of advantages in compound and alloy deposition, including low temperature deposition, relative low cost, uniform coverage on complicated surfaces, and is scalable for large area deposition. Thermal ALD discussed in this paper is a vacuum based deposition method, in which each precursor in vapor gas form is introduced into the vacuum reaction chamber sequentially and repeatedly, and a monolayer film deposit is formed via a surface saturating chemical reaction in each ALD monolayer deposition cycle. ALD technology can precisely control the film layer thickness, stoichiometry, composition, uniformity, and produce sharp interfaces. Due to its surface saturating properties, ALD can also be used to deposit conformal films onto very complex 3-D structures and on the inside of cavities and negative slope surfaces [52,53]. It is also possible to generate reproducible and well defined nanolaminate structures, which is exactly what is required for the deposition thermoelectric films on patterned substrates, for example porous phononic crystal (PnC) substrates [1]. For these reasons, the ALD technique is ideally suited for the deposition of thermoelectrical materials on complex substrates in a controllable manner, which will be elaborated in detail in this paper. The ALD technique has also found widespread application in the deposition of conformal complex and doped system. Binary metal oxide compound synthesis in ALD, such as ZnO, Al_2_O_3_, ZnS, is typically formed by repeating binary cycles consisting of one precursor dose and followed by the other co-reactant dose. For doped structures or ternary compound synthesis in ALD, such as aluminum-doped ZnO (AZO), or perovskite oxides, the most common approach is alternatively depositing multiple cycles of two binary cycles, interspersed with specific delta doping layers and subsequently followed by post annealing steps to achieve the desired electrically activated doped crystal structure. The advantage of ALD [54,55] for the synthesis of doped, ternary, and quaternary materials by ALD [56], as well as functional perovskites by ALD [57], have been well reviewed previously, and will not be covered in this paper. Among the drawbacks of ALD are the relatively lower deposition rate, limited types of precursors available (where some maybe costly), relatively lower film crystal quality and purity because of moderate vacuum level and low deposition temperatures. However, it is precisely the low temperature deposition, the precise thickness control and absolute conformality properties that predestine ALD for nanolaminate deposition, and depositions on complex or soft substrates, which will be discussed in the following sections.

## 3. Nanostructured Thermoelectrical Materials in zT Improvement 

### 3.1. Phononic Crystal

**Figure 5:**
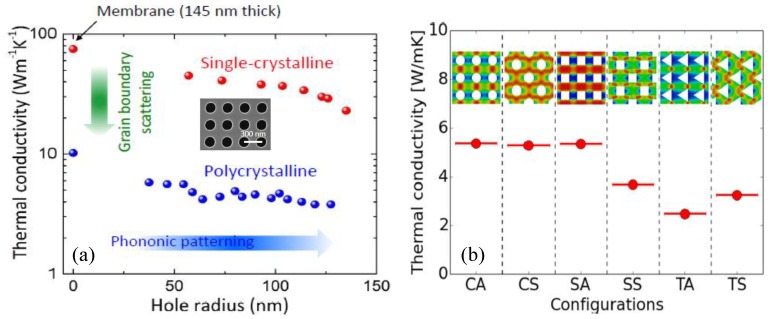
(**a**) Thermal conductivity of a Phononic Crystal (PnC) vs. the radius of the holes in porous templates. The inserted image is a schematic diagram of a PnC with hole-center distance of 300 nm [63]. (**b**) Theoretical simulation of the dependence of thermal conductivity on the configurations of variously shaped pores in porous membranes [67].

As mentioned in the introduction, the reduction of thermal conductivity is a key strategy to further improve thermoelectric materials. Phononic crystal (PnC) nanostructures in thermoelectric materials have recently caught researchers’ attention because of their lower thermal conductivity compared to non-patterned thermoelectric samples, due to phonon-boundary scattering [58,59,60,61,62]. The work from the University of Tokyo has produced 2-D phononic crystal nanostructure using single crystalline or polycrystalline porous silicon membranes. PnC structures were fabricated via electron beam lithography using a reactive ion etching coupled plasma system, with SF_6_/O_2_ gas as etchant [63]. The team of Prof. Masahiro Nomura at the University of Tokyo studied the thermoelectrical properties of PnCs with different hole radii, crystalline structures and doping types [58,59,63,64]. Figure 5a shows experimental results for the thermal conductivity of PnCs made from single crystalline Si and polycrystalline porous Si membranes, as a function of the radius of pores. Notably, 2-D PnCs made from polycrystalline porous Si exhibit lower thermal conductivity, which is attributed to the grain boundaries scattering of thermal phonons with relatively short mean free path (MFP), and periodic patterns scattering phonons with relatively longer MFPs. The thermal conductivity of PnCs was dramatically reduced as the hole radius increased, resulting from backscattering of phonons by the internal walls of the porous structures. The effect of PnCs on the thermal conductivity were also observed in both p-type and n-type doped PnCs structures [64]. The electrical conductivity of PnCs also exhibits a reduction, as the hole radius of PnCs is increasing, but it is less sensitive to the hole radius compared to thermal conductivity. Hence, the authors observed a zT enhancement factor of two and four in p-type and n-type PnCs, respectively. 

In addition, the geometric arrangement of pores in porous membranes plays a role in the reduction of thermal conductivity [44,65,66]. Porous silicon template substrates and thin porous Si membranes are a relatively novel and versatile material, with tunable properties related to their porosity and porous geometry. The thermal conductivity of porous templates is tunable by modulating the porous configuration (staggered or aligned) [65], and by the geometrical shape of the pores (circular or square or triangular), which was demonstrated in theoretical simulations by the group of G. Romano at MIT [67] and the periodicity [58]. Figure 5b displays the dependence of thermal conductivity of porous Si on the shape and geometrical arrangement of the pores. It has been proven by theoretical and experimental work that phonon-boundary scattering in porous materials contributes significantly to reducing phonon thermal conductivity, up to two orders of magnitude compared to bulk materials [16,67]. Therefore, phonon-boundary engineering tuning thermal transport in nanostructured materials is a powerful approach to develop high-efficiency thermoelectrical materials. Depositing conformal thermoelectric nanolaminate films on nano-patterned porous substrates turns out to be a successful approach to improve the Seebeck coefficient and the figure of merit zT of TE materials by significantly decreasing the thermal conductivity, which was predicted by simulation work and was subsequently proven experimentally, and will be discussed in detail in later sections. 

### 3.2. Lead Chalcogenide Based ALD Nanolaminate on Porous Templates

**Figure 6:**
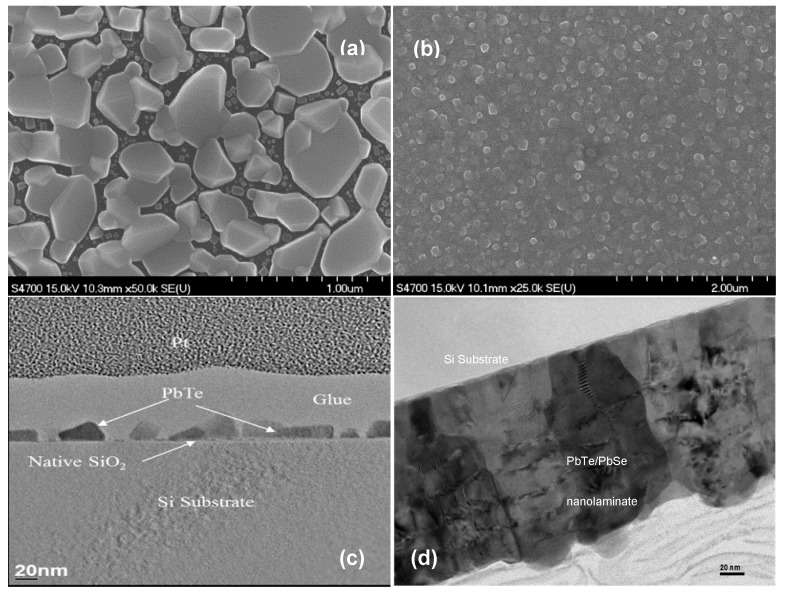
FE-SEM micrographs of (**a**) 1000 ALD deposition cycles of PbTe film without pre-treating the Si substrate resulting not in a dense film, (**b**) compact PbTe/PbSe (10/10 nm) nanolaminate film grown on hydroxyl OH^-^ terminated Si substrate [1], (**c**) TEM cross-sectional images highlighting the onset of the early stages of nucleation of an ALD deposited PbTe film of 700 ALD deposition cycles grown at 170 °C on Si substrate [72], (**d**) featuring thicker PbTe/PbSe (10/10 nm) nanolaminate layers, when all the initial nuclei have finally coalesced into continuous compact layers grown on a Si substrate at 150 °C [73].

**Figure 7:**
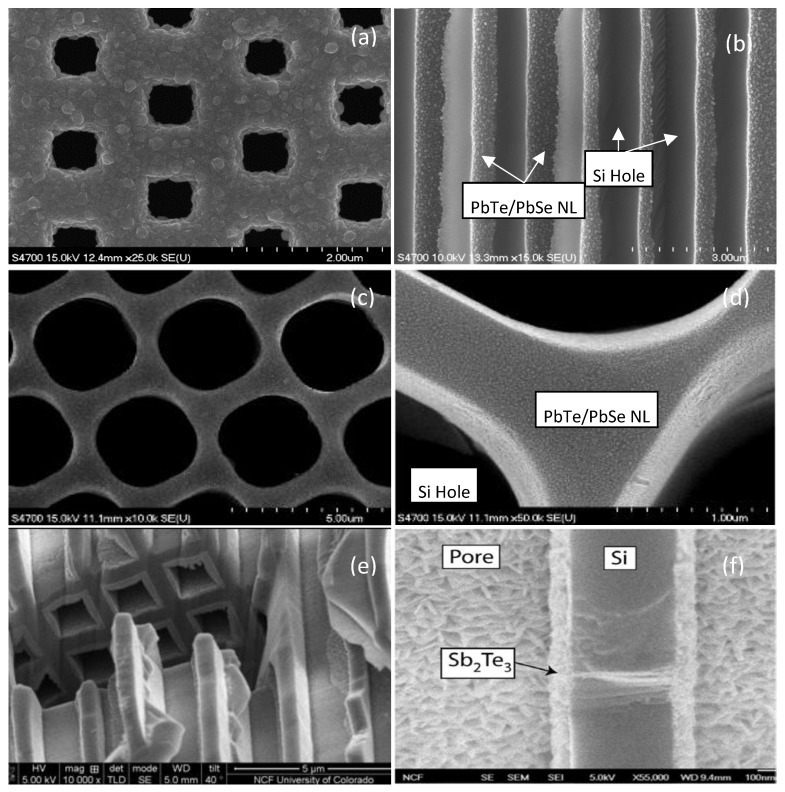
**(a)** FE-SEM images of PbTe/PbSe (10/10 nm) nanolaminates grown on porous Si substrate with misaligned staggered square pores of 700 nm × 700 nm size, which are horizontally spaced 1.72 μm apart, and 0.6 μm vertically apart [1]. (b) cross-sectional images of PbTe/PbSe nanolaminates with period of 10 nm [73]. (**c**) and (**d**) SEM image of ALD deposited PbTe/PbSe on porous Si templates [68]. (**e**) and (**f**) are ALD deposited Sb_2_Te_3_ film on porous Si templates with square shaped pore structures from the team at MicroXact Inc. [71].

PbTe and PbSe are well-known traditional thermoelectrical materials. Both bulk alloy compound and nanostructured PbTe/PbSe have been investigated over the decades. Nanostructured PbTe/PbSe film can be deposited by MBE [8,48] and E-ALD [68,69]. To the best of our knowledge, vacuum based thermal ALD technology was first applied for the synthesis of PbTe/PbSe nanolaminate structures in our work [1,70]. Both ALD synthesized PbTe and PbSe films exhibit a polycrystalline structure with granular film morphology. The polycrystalline nature of these thermoelectric films provides a sufficient number of grain boundaries to scatter phonons, which exactly meets our requirements. The initial experimental results indicate the growth of PbTe and PbSe films by ALD and follow the Volmer–Weber island growth mode [70]. However, the grains are isolated in random size and orientation, and do not fuse together no matter how many ALD cycles were repeated, which can be seen from the FE-SEM surface morphology image in Figure 6a and the TEM cross-sectional images in Figure 6c. The nucleated PbTe islands are isolated and exhibit random size and orientation, nucleating in a typical Volmer–Weber type of island growth mode. Achieving uniform and complete coverage of the sample surface with compact dense ALD films would be the crucial first step to fabricate nanolaminate thermoelectric structures. The isolated granular surface morphology of ALD PbTe and PbSe results from the fact that atom-to-surface reactions dominate in the ALD growth of PbTe and PbSe. By pre-treating the substrates with H_2_O_2_/H_2_SO_4_ (volume ratio: 1:4) for five min, a saturated OH^-^ hydroxyl surface termination was achieved, which optimized the growth conditions to enhance the chemisorption of the Se and Te precursors on the substrate. As a direct benefit, the initially isolated nucleated PbSe islands finally coalesced and combined tightly into continuous compact and dense thermoelectric PbTe and PbSe films, shown as FE-SEM surface morphology image in Figure 6b and the TEM cross-sectional images in Figure 6d. The ALD deposition process for PbTe/PbSe nanolaminate films is described in detail in our previous paper [1]. Figure 7a–d show SEM micrographs of ALD deposited PbTe/PbSe nanolaminates on porous Si templates from our work at Old Dominion University, and Figure 7e,f show SEM images of ALD deposited Sb_2_Te_3_ inside the rectangular shaped microporous Si pores, reported from the work of our collaborator V. Kochergin’s group, at MicroXact Inc. [71] Both works exhibit complete conformal coverage with nanocrystalline thermoelectric ALD films on porous silicon template surface, completely coating the inside walls of every pore structure, which further highlights the significant advantage of ALD technology for coatings of complex 3-D surface morphologies and high aspect ratio pores in porous templates. To our best knowledge, there is no other research work on ALD deposited thermoelectrical nanolaminates on porous templates. Our previous work demonstrates both ALD deposited PbTe and PbSe films are slightly non-stoichiometric being Pb rich, which indicates the PbTe and PbSe films are n-type semiconductors, and electrons are the majority charge carriers [1], shown as energy dispersive X-ray spectroscopy analysis EDS result in Figure S1 and Table S1 in Supplementary Materials. This was further corroborated by subsequent Seebeck coefficient measurements.

**Figure 8:**
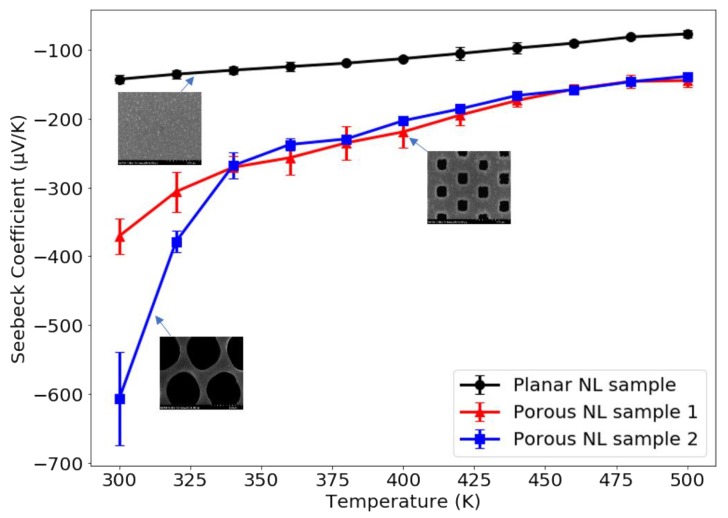
Plot of Seebeck coefficient as a function of temperature comparing ALD PbTe/PbSe (10/10 nm) nanolaminates deposited on planar Si substrates, versus ALD deposition on microporous silicon templates with small square shaped pores, and versus porous silicon templates with thinner pore wall and larger pore diameter, measured by MMR Seebeck measurement system. The data were extracted from previous published works of Old Dominion University [1,73].

It is noteworthy that the in-plane Seebeck coefficient for our PbTe/PbSe nanolaminate structure measured on square shaped porous Si membranes is about 2.5 times larger compared to the value obtained for non-structured PbTe/PbSe nanolaminates on planar bulk Si substrates. Figure 8 displays the temperature dependence of the Seebeck coefficient for PbTe/PbSe nanolaminates, grown on planar silicon wafers and porous silicon templates. The maximum Seebeck coefficient of PbTe/PbSe nanolaminates on round-shaped porous Si membranes reached to −574.24 ± 20.79 µV/K at 300 K, which is about four times higher than that of the planar sample. The enhanced Seebeck value is attributed to the presence of the regular array of etched out pores, which provides an additional periodic structuring of the nanolaminate composite sample, while the high porosity of the porous Si template simultaneously reduces the thermal conductivity, with more air than Si remaining in the template structure. 

The Seebeck coefficient of all the thermoelectric ALD samples under test reaches a maximum value around room temperature. Our measured temperature dependence of the Seebeck coefficient is different from the reported value in reference [74], where the maximum Seebeck coefficient occurs at 560 K. One of reasons for the discrepancy may be attributed to different thermoelectric film properties, due to different deposition techniques. It is believed that the maximum Seebeck coefficient occurs when intrinsic conduction begins. However, our non-doped PbTe and PbSe films evidently have a lower carrier concentration than the doped films in the literature, which consequently results in the onset of intrinsic conduction at a lower temperature [75]. For the Seebeck coefficient measurements in the direction vertical to the surface, our collaborator at MicroXact, Inc. measured similar trends and found that the PbTe/PbSe nanolaminate structure grown on round-shaped porous silicon membranes exhibits even higher absolute Seebeck coefficients, with values of 78670 ± 15540 µV/K in the vertical direction, compared with the thermoelectric nanolaminate structure grown on a planar bulk silicon wafer (250 ± 27 µV/K), shown as Figure S2 in the Supplementary Materials [68].

This porous Si membrane was fabricated by removing the Si from the backside with KOH etching, until the pores are reached and opened from the backside. From the results of our Seebeck measurements in both the horizontal and vertical directions, the low dimensional ALD thermoelectric nanolaminate structures synthesized inside the pores of porous silicon membranes and conformally coating all inside pore walls exhibit higher Seebeck coefficients than previously reported values [74,76]. This indicates that the added periodic surface structuring and the dimensions of the pore diameter and pore walls resulting from the photo-lithographically defined regular pore pattern of the porous Si membrane constitutes another crucial parameter to aid in further improving the Seebeck coefficient of TE materials. This is attributed to the lower thermal conductivity κ in porous Si membrane structures. The porous silicon membrane we used exhibits a staggered square porous configuration, which theoretically yields the lowest thermal conductivity compared to other porous configuration arrangements [67]. This is further confirmed in our thermal conductivity measurements, which are discussed in detail in later sections. Since the temperature gradient is the driving force for charge carriers moving from the hot side to the cold side, such a temperature gradient will be more lasting in a material with lower thermal conductivity, so that more charge carriers accumulate at the cold side, leading to a larger voltage difference between the two sides, and this consequently results in a larger Seebeck coefficient. Another contributing factor is the observed reduction in electrical conductivity σ in our patterned samples, which can be attributed to electron scattering by the sidewalls of the pores. Since σ=1/ρ=neμ, this in term leads to a reduction in charge carrier density n. According to the Mott relation shown in Equation (4), the Seebeck coefficient is inversely proportional to n. Therefore, this observed reduction in *n* contributes to the significantly increased Seebeck coefficients measured in PbTe/PbSe nanolaminate layers on porous Si templates. 

**Figure 9:**
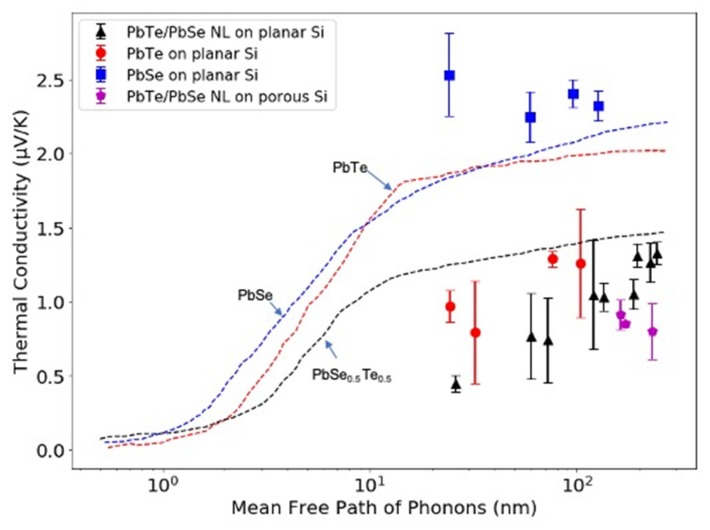
The cumulative thermal conductivity of the PbTe-PbSe, with respect to mean free path of phonons at 300 K, shown as dotted lines. The data were extracted from ref [77] and are based on theoretical calculations. The superimposed symbols with error bars were individual experimental thermal conductivity measurements of our ALD PbTe, PbSe, PbTe-PbSe nanolaminates, grown on planar and porous silicon substrates with respect to their total thickness. The thermal conductivity data were measured by time domain thermo-reflectance (TDTR) at University of Virginia (UVA). The data were extracted from ref [78] and [73].

The porous nanolaminates also simultaneously exhibited a significant reduction in thermal conductivity. Figure 9 displays the relationship between thermal conductivity and the phonon mean free path/thickness of PbTe and PbSe films. The dotted lines are cumulative thermal conductivity with respect to phonon mean free path at room temperature, that were calculated by Tian’s group at MIT [77]. For comparison, the overlaid dot symbols were individual measurements of cross-plane thermal conductivity of PbTe, PbSe and PbTe/PbSe nanolaminates, grown on planar and porous silicon substrates with respect to their total thickness, reported by our collaborators Mallory E. DeCoster et al. at the University of Virginia (UVA) [78]. In general, PbTe/PbSe nanolaminates exhibited lower thermal conductivity, compared to parent PbTe and PbSe films. The simulation work from Tian et al. suggested that phonons with MFP ranging from 1–10 nm contributed to the majority of heat transport in PbTe, PbSe and their alloys. The experimental work from Mallory E. DeCoster et al. indicated that the thermal conductivity of the nanolaminate PbTe/ PbSe films started to exhibit size effects when the thickness of the films was less than 121 nm. Increased phonon scattering was believed to be attributed to compositional points defects and interface boundaries between PbTe and PbSe layers, or other scattering defects like dislocations. The Volmer–Weber growth mode of ALD deposited PbTe and PbSe results in a further reduction in thermal conductivity, especially as film thickness is reduced [78]. Additionally, the ALD nanolaminates grown on porous templates show slightly further reduction in thermal conductivity compared to the case of the planar nanolaminates samples. In addition, the ALD nanolaminates exhibit a lower electrical conductivity compared to non-patterned nanolaminates, which is similar to reported literature work [64]. The reduction in electrical conductivity in patterned samples is attributed to the electron scattering by the sidewall of the pores. The zT value of the nano-structured porous sample was enhanced by a factor of up to three at 500 K [73]. 

### 3.3. ZnO-Based ALD Themoelectric Nanolaminates 

ZnO is a great semiconductor material that has been widely applied in transparent thin film transistors, photodetectors, light-emitting diodes, gas sensor, etc., due to its wide and direct band gap, suitable intrinsic n-type doping, and high electrochemical stability. Doping substitutional Zn sites with dopants such as Al, Ga, and Fe can improve the electrical properties of ZnO by tuning its carrier concentration and band gap. Researchers also investigated the application potential of doped ZnO in thermoelectrics. Won-Yong Lee etc. at Chung-Ang University, S. Korea, investigated cross-plane thermoelectric properties of ALD deposited Al_2_O_3_/ZnO (AO/ZnO) superlattices over the temperature range of 300 K to 500 K [79]. The Seebeck coefficient of undoped ZnO and doped AO/ZnO superlattices are −171 μV/K and −179 μV/K, respectively. But the Seebeck coefficient of doped AO/ZnO superlattice samples increased ~337% more rapidly as the temperature increased and trended to become larger than the undoped ZnO sample [79]. The thermal conductivity of the AO/ZnO superlattice is 0.31 W/mK, which is about 1233% lower than that of the undoped ZnO sample. Phonon scattering at AO and ZnO interfaces and defect scattering were believed to be contributing to thermal conductivity reduction. Consequently, doped AO/ZnO superlattices achieved a maximum zT of 0.45 at 500 K, which is approximately 2650% higher than the zT of undoped ZnO sample. Gallium-doped ZnO films were reported, that achieved a maximum power factor of 0.66 mW/mK^2^, and a Seebeck coefficient of 60 μV/K with 1% Ga doping at room temperature [69]. 

Shuankui Li’s group at Peking University, Shenzhen Graduate School, China, reported thermoelectric improvement in ALD ZnO coating on Bi_2_Te_2.7_Se_0.3_ (BST) by effective atomic interface engineering. BST nanosheets were synthesized through a simple solution-based approach [80]. BST powder was synthesized by solution-based approach, followed by applying ALD ZnO coatings on BST powder. Then, the ALD ZnO coated BST powder was pressed into pellets by hot pressing at high temperature. Taking advantage of ALD’s precise thickness control and conformal coverage, ultra-thin ZnO layers were introduced onto the BST matrix, which generates potential barriers and grain boundaries. The thickness of the ALD ZnO layer and the grain size of BST was optimized to extensively scatter phonons, while affecting less the electron transport, which consequently resulted in improving the Seebeck coefficient and the figure of merit zT. The authors observed Te diffusion into the ALD ZnO layer after annealing at high temperature, which forms Te nanodots at the ZnO layer. The nanodots, in a small size of 2 nm, were believed to serve as an electrical conduction bridge between two adjacent BST grains, and also contribute to enhancing the Seebeck coefficient, due to quantum confinement effects. The authors reported 80% zT enhancement in ALD ZnO coated BST structure, compared to pure BST.

### 3.4. Hybrid Organic-Inorganic Superlattice Structures

In addition to traditional inorganic thermoelectrical materials, the thermoelectric properties of organic materials have also been widely investigated by researchers because of their intrinsically low thermal conductivity, light weight, and non-toxicity. Organic materials, such as CNT [81], PEDOT:PSS [82], Guest loaded Metal Organic Framework (MOF) [83,84,85], etc., have been reported for their potential applicability in thermoelectrics. Hybrid inorganic-organic structures were successfully synthesized. Chunlei Wan et al. at Nagoya University reported a hybrid inorganic-organic superlattice structure of TiS_2_ layers intercalated with organic molecules [(hexylammonium)_x_(H_2_O)_y_ (dimethylsulphoxide)_z_]) [86], fabricated by ALD/MLD method, exhibiting a low thermal conductivity of 0.12 Wm^−1^K^−1^ and a figure of merit of 0.28 at 373 K. The superlattice engineering was believed to be the main reason for reducing the thermal conductivity of the structure. Antti J. Karttunen et al. at Aalto University, Finland reported thermoelectric properties of flexible thermoelectrical ZnO-organic superlattices grown on cotton Textile substrates by ALD/MLD [87]. The substrate was pre-deposited with a thin Al_2_O_3_ seed layer. The authors at Aalto University observed a Seebeck coefficient enhancement in ZnO-HQ (hydroquinone) superlattice samples. The Seebeck coefficient of the superlattice sample is up to −411 µV/K at room temperature, which is about 2.8 times higher than that of pure ZnO film. It is difficult to accurately measure the thermal conductivity of hybrid superlattice samples on flexible substrates, but there are a few research works that investigated thermal conductivity of similar hybrid inorganic-organic superlattices on planar substrates [88,89]. These reports indicated that the introduction of organic layers in the hybrid superlattice structures contribute to reducing the cross-plane thermal conductivity up to two orders of magnitude [87]. The use of highly flexible substrates demonstrated the feasibility of applying hybrid superlattice structures for flexible or wearable thermoelectrics. Kyung Tae Kim et al. at the Korea Institute of Materials Science [90] investigated the thermoelectric properties of CNT/Bi_2_Te_3_ composite powders, showing a maximum zT of 0.85 at 473 K and a thermal conductivity of 0.6 Wm^−1^K^−1^ at 300 K, which is 40% lower compared to the Bi_2_Te_3_ matrix at the same temperature. The introduction of organic CNTs generated numerous interfaces in a Bi_2_Te_3_ matrix that enhanced phonon scattering, and therefore reduced thermal conductivity and yielded high zT values [90].

## 4. Concluding Remarks and Future Outlook

This short review is summarizing recent progress on thermoelectric materials improvement achieved by ALD deposited nanolaminates and applying PnCs structures for phonon engineering. The thermal conductivity of PnCs structures were significantly reduced, as hole size of porous templates increased from 0 nm to 120 nm. p-type and n-type PnCs exhibited 59% and 84% reduction in thermal conductivity, respectively. The zT enhancement was observed in both p- and n-type PnCs, where the enhancement factor was two and four, respectively. The greater zT enhancement was attributed to stronger neck effect and spread distribution of the phonon MFP. This work indicates that nanopatterning is a promising approach for thermoelectric materials improvement. Theoretical research work from the literature indicates that the pore shape and pore arrangement of porous templates can be optimized for lower thermal conductivity. Pursuing a strategy of nanopatterning for phonon engineering, the team at Old Dominion University (ODU) investigated the thermoelectric properties of ALD deposited PbTe/PbSe nanolaminates on porous silicon templates. ALD thermoelectric nanolaminates on porous templates exhibited an improvement of 2.5 times in the Seebeck coefficient, plus a 45% reduction in electrical conductivity, and a 38% reduction in thermal conductivity compared to planar ALD nanolaminate samples, which consequently resulted in a zT enhancement for the nanolaminate sample by a factor of up to three at 500 K. The work at ODU demonstrates the feasibility of applying nano patterned TE elements for TE devices for an improved performance. The superior conformality of the ALD film synthesis is critical for achieving a complete coverage and uniform coating with thermoelectric nanolaminates on the interior walls of all pores in porous templates. Furthermore, the excellent thickness control of ALD allows for ultra-thin layer deposition and composition control (e.g. better for adjusting doping concentration). A significant zT improvement was observed in AO/ZnO superlattice, achieving a zT of 0.45 at 500 K, which is about 2650% higher compared with undoped ZnO samples. In addition, hybrid organic-inorganic superlattice structures also were reported to exhibit a sizeable thermoelectric improvement compared to pure organic or inorganic bulk parent materials.

The successful deposition of nanostructured thermoelectrical materials on planar, porous and flexible templates highlights the crucial advantages of ALD, including low temperature deposition, absolute conformality, superior thickness control, enabling the deposition of ultra-thin superlattice nanostructures and ALD doped composite films. ALD contributes significant technical capabilities to modern thermoelectric materials improvement. However, there are still some challenges to overcome. ALD requires precursors that must be volatile but are not subject to decomposition, which limits the availability of thermoelectric materials that can be deposited by ALD. ALD deposited thermoelectrical materials, that have been reported for the time being, include primarily lead chalcogenides, such as Bi_2_Te_3_, Sb_2_Te_3_, PbTe, PbSe, and their layered structures, as well as ZnO based superlattices and their doped structures. Lead chalcogenides are easily oxidized and degraded at high temperature. The moderate vacuum level in thermal ALD deposition reactors may decrease the quality and purity of the films. The present reported work indicates that ALD deposited PbTe/PbSe nanolaminates on porous silicon templates exhibit a zT value improvement of up to a factor of three. However, the zT value of the ALD nanostructures is lower compared to the reported zT of PbTe/PbSe based nanostructures deposited by MBE. This difference can be attributed to the lower carrier concentration in undoped structures, and the lower quality and onset of oxidation in the thermoelectric films obtained by ALD deposition. It is noteworthy to add that MBE is not a contender at all for industrialization, while ALD is already a proven high-volume fabrication technology in the microelectronics industry. Doping in ALD PbTe/PbSe nanolaminate structures can be further investigated to optimize any thermoelectric improvements. A higher vacuum level may be needed for ALD deposition of lead chalcogenides based thermoelectrical materials to avert oxidation. 

In summary, ALD is an outstanding thin film deposition technique for the synthesis of nanostructured thermoelectrical materials. Nanopatterning is a promising approach and worth investigating for the improvement of thermoelectric properties by enabling structuring of thermoelectric materials on the length scale of the mean free path of phonons. 

## Supplementary Materials

The following are available online at https://www.mdpi.com/1996-1944/13/6/1283/s1, Figure S1: Energy dispersive X-ray Spectroscopy analysis (EDS) of a PbSe film deposited with 1000 ALD cycles and of a PbTe samples grown with 1000 ALD deposition cycles, Figure S2: Voltage and temperature response to infrared heat pulse at room temperature for PbTe/PbSe (10 / 10 nm) (a) grown on planar Si substrates and (b) on porous Si templates, Table S1: The composition of lead, tellurium and selenide in PbTe and PbSe films.
