# Supplementary material for: Advances in Atomic Layer Deposition (ALD) Nanolaminate Synthesis of Thermoelectric Films in Porous Templates for Improved Seebeck Coefficient
Source: Materials (Basel). 2020 Mar 12;13(6):1283. doi: 10.3390/ma13061283 (PMC7143041; doi:10.3390/ma13061283)
Supplement: Supplementary file 1 [file materials-13-01283-s001.pdf]

materials

Supporting Information for Advances in Atomic Layer Deposition (ALD) Nanolaminate Synthesis of Thermoelectric Films in Porous Templates for Improved Seebeck Coefficient

Xin Chen [a,b], Helmut Baumgart [a,b]

a Department of Electrical and Computer Engineering, Old Dominion University, Norfolk, Virginia 23529, USA

b Applied Research Center at Thomas Jefferson National Accelerator Laboratories, 12050 Jefferson Avenue, Suite 721, Newport News, Virginia 23606, USA

The composition and stoichiometry of the samples were observed by energy dispersive X-ray spectroscopy analysis (EDS), shown as figure s1. The result from EDS reveals the chemical element composition in PbTe and PbSe films grown at 150 ˚C with 1000 ALD deposition cycles. Table s1 lists the element compositions in PbTe and PbSe samples. Both PbTe and PbSe are slightly non-stoichiometric being Pb rich, which indicates the PbTe and PbSe films are n-type semiconductors, and electrons are the charge carriers.

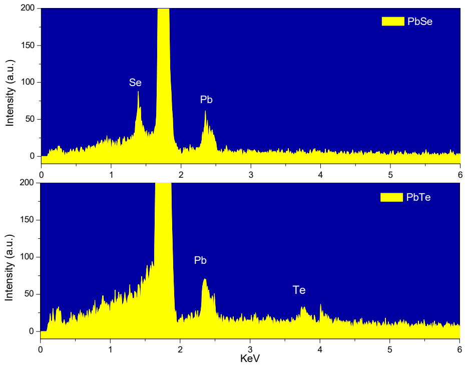

Figure S1. Energy dispersive X-ray Spectroscopy analysis (EDS) of a PbSe film deposited with 1000 ALD cycles and of a PbTe samples grown with 1000 ALD deposition cycles [1].

The Seebeck coefficient of PbTe/PbSe nanolaminates grown on porous silicon templates along cross-plane direction was measured by IR Seebeck Characterization Systems at MicroXact, Inc. Figure s2 displays voltage and temperature response to infrared heat pulse at room temperature for PbTe/PbSe nanolaminates grown on planar and porous silicon templates.

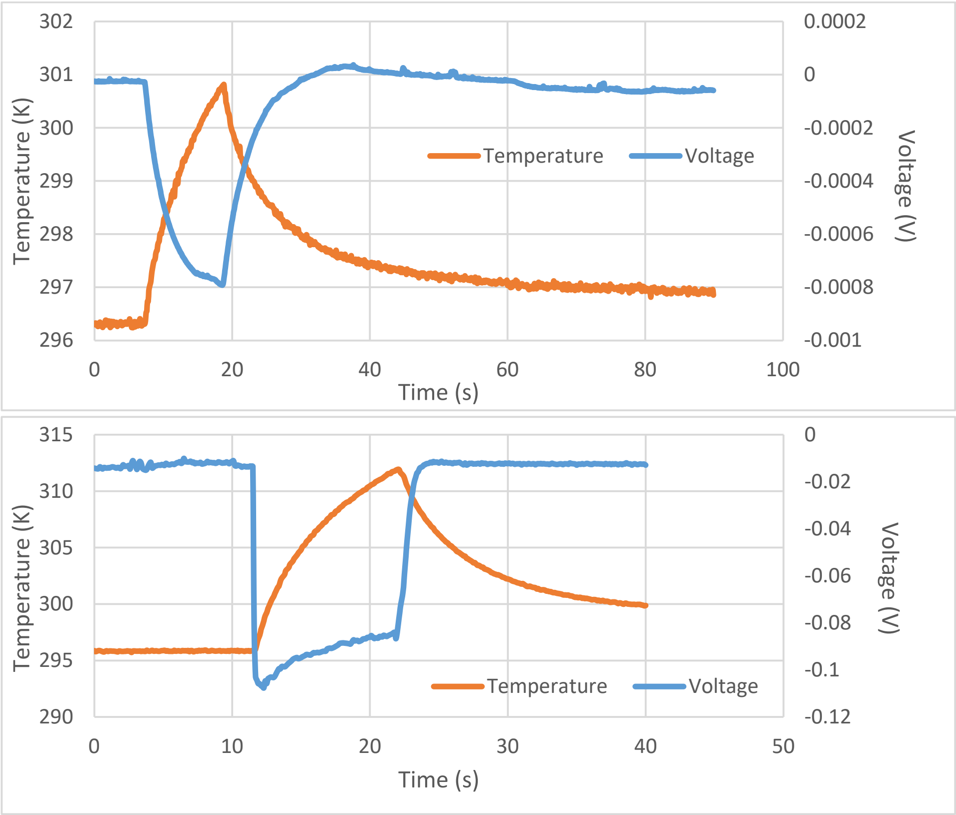

Figure S2. Voltage and temperature response to infrared heat pulse at room temperature for PbTe/PbSe (10 / 10 nm) (a) grown on planar Si substrates and (b) on porous Si templates [2].

Table S1. The composition of lead, tellurium and selenide in PbTe and PbSe films [1]

Samples	Pb (Weight %)	Te (Se) (Weight %)	Pb (Atom %)	Te (Se) (Atom %)
PbTe (1000cys-170 ˚C)	6.53±1.61	1.98±0.22	0.86±0.21	0.42±0.05
PbSe (1000cys-170 ˚C)	12.07±0.81	3.68±0.26	1.88±0.13	1.5±0.1

1. Chen, X., et al., *Seebeck Coefficient Enhancement of ALD PbTe/PbSe Nanolaminate Structures Deposited inside Porous Silicon Templates.* ECS Journal of Solid State Science and Technology, 2016. **5**(9): p. P503-P508.
2. Chen, X., *Enhancement of Thermoelectric Properties of ALD Synthesized PbTe and PbSe by Phonon Engineering*. 2017: Old Dominion University.
